# Mesocosms Reveal Ecological Surprises from Climate Change

**DOI:** 10.1371/journal.pbio.1002323

**Published:** 2015-12-17

**Authors:** Damien A. Fordham

**Affiliations:** The Environment Institute and School of Biological Sciences, The University of Adelaide, Adelaide, South Australia, Australia

## Abstract

Understanding, predicting, and mitigating the impacts of climate change on biodiversity poses one of the most crucial challenges this century. Currently, we know more about how future climates are likely to shift across the globe than about how species will respond to these changes. Two recent studies show how mesocosm experiments can hasten understanding of the ecological consequences of climate change on species’ extinction risk, community structure, and ecosystem functions. Using a large-scale terrestrial warming experiment, Bestion et al. provide the first direct evidence that future global warming can increase extinction risk for temperate ectotherms. Using aquatic mesocosms, Yvon-Durocher et al. show that human-induced climate change could, in some cases, actually enhance the diversity of local communities, increasing productivity. Blending these theoretical and empirical results with computational models will improve forecasts of biodiversity loss and altered ecosystem processes due to climate change.

## Abstract 2

This Primer uses two recent studies to examine how mesocosm experiments are improving our understanding of the ecological consequences of climate change on species’ extinction risk, community structure, and ecosystem function.

## Introduction

Models forecast that human-induced climate change is likely to cause extinctions and alter diversity patterns, directly and in synergy with other drivers of global change (habitat destruction, overexploitation, and introduced species), but the range of estimates for its total impact remains worryingly large [1]. A more evidence-focused approach to climate impacts research is required to gain deeper insights into the likely effects of shifts in climate on biodiversity over the coming decades to centuries—and, through these insights, to design effective adaptation strategies that mitigate climate-driven biodiversity loss [2].

Data from natural history collections, repeated surveys, and other monitoring activities continue to be used to study biotic responses to 20th century climate change [3]. Although these studies have increased our knowledge of how species can vary their phenologies, distributions, abundances, and phenotypes in response to climate change, linking these observations to long-term effects on species’ persistence, community structure, and ecosystem function has proven difficult [4]. This is partly because resurvey and monitoring studies inevitably focus on near-term outcomes, meaning that they are typically unable to consider species responses to large shifts in climate—those similar in magnitude to those predicted for the 21st century and beyond [5]. Another problem is the lack of field-based experimental approaches (e.g., translocation experiments) in climate ecological research, which can directly attribute ecological mechanisms to biotic responses to different climatic conditions using cause-effect relationships [6].

In contrast, laboratory microcosm (or small-scale field experiments) and larger scale mesocosm experiments allow rigorous testing of climate impacts on populations and communities, improving our theoretical understanding of ecological responses to likely climate shifts [7]. They do this by providing tractable yet ecologically realistic bridges between simplified experimental conditions and the real world [8]. For example, warming experiments have provided important stimulus for further research on trait plasticity and resilience to climate change [9], the importance of synergies among drivers of endangerment [10], the role of temperature and habitat isolation on community composition [11], and the impact of global change on ecosystem function [12]. As ecological climate change research moves to increasingly more mechanistic approaches, experiments are today being constructed at ever larger scales with higher biocomplexity, with the ultimate aim being to parameterize, test, and refine models that accurately predict the effects of climate change on biodiversity (Box 1) [13]. Two papers recently published in *PLOS Biology* highlight why mesocosm experiments provide such powerful tools for identifying the ecological processes that drive population- and community-level responses to climate change and for testing fundamental principles of ecology.

**Box 1. Integrating Mesocosms with Ecological Models to Improve Predictions of the Ecological Consequences of Climate Change**

**Fig 1:**
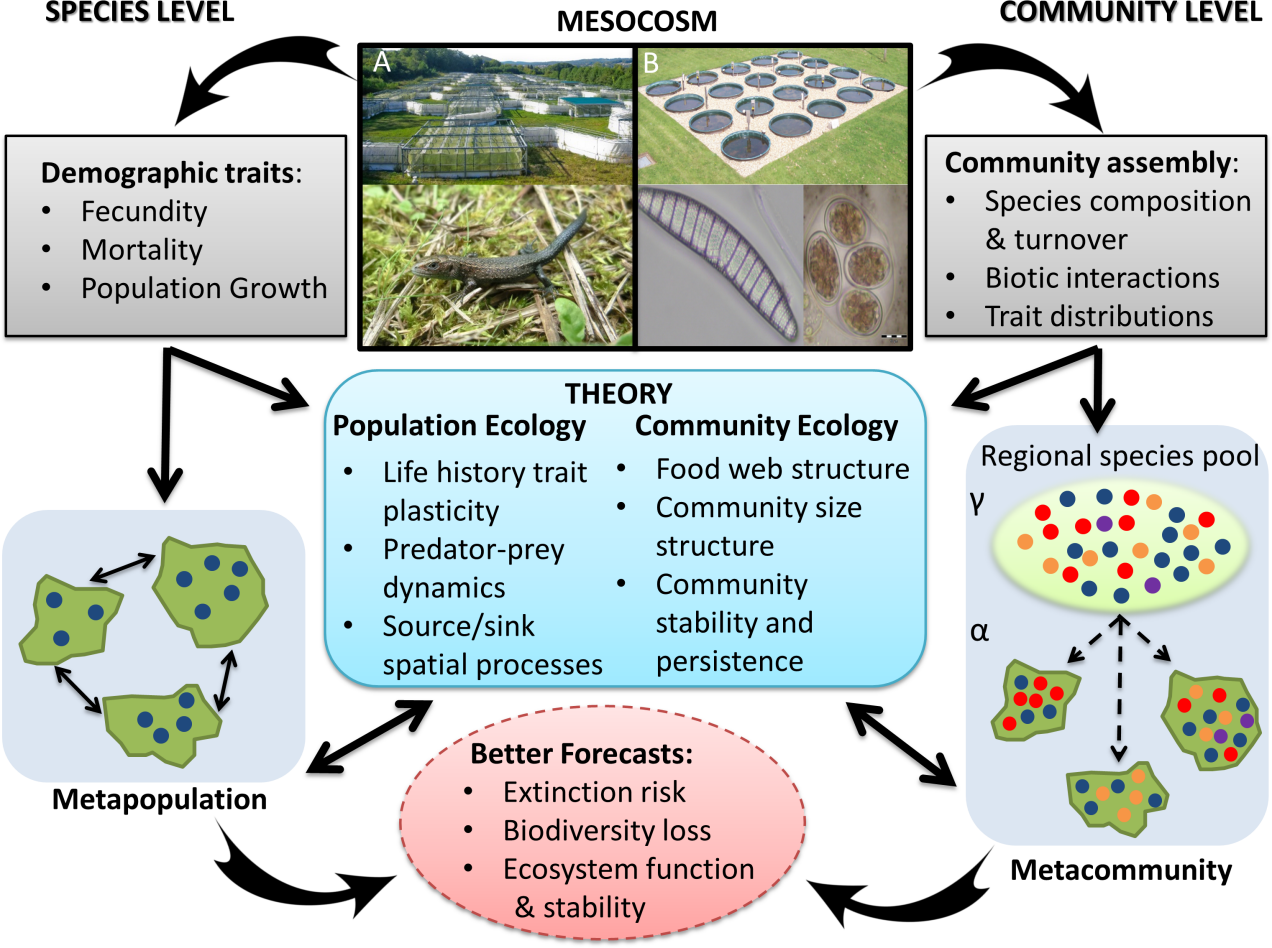
Using mesocosms to parameterize and refine climate-biodiversity models. Mesocosm experiments can be used to improve predictions of the impact of climate change on individual species and whole communities by parameterizing metapopulation and metacommunity models and by testing and refining population and community ecology theory. The figure is described in detail in Box 1. Photos in panel A show the Metatron infrastructure used to study demographic responses to warming among common lizards (*Zootoca vivipara*) [14]. Panel B shows the outdoor mesocosm experiment used to determine the impact of warming on the metacommunity dynamics of phytoplankton [20].

Mesocosms have a central role to play in predicting the impact of climate on different ecological levels, ranging from individual species to whole communities (and potentially to entire ecosystems). At the species level, they enable the effect of global warming on demographic traits (fecundity, mortality, density dependent population growth rate, etc.) to be directly estimated. This information can be integrated into population models to determine risk of extinction in the absence of immigration and emigration (Fig 1) [14]. Data on species’ physiological tolerance from mesocosm experiments can also be coupled with spatial geographic information system (GIS) layers of present-day and likely future climatic conditions to predict the potential range of a species [15]. Using this information in metapopulation models to define dynamic patch structures improves estimates of extinction risk from climate change, by accounting for important spatial and demographic processes and their interaction [16]. If natal dispersal is not estimated in the mesocosm experiment, field-based or allometric estimates can be used in the metapopulation model. Mesocosm experiments can also be used to directly improve our understanding of key principles of population ecology, including the importance of plasticity in life history traits and predator–prey dynamics on persistence (Fig 1). Furthermore, metapopulation and demographic models, coupled to mesocosm experimental data, can be used to test and improve theoretical expectations. Together this will lead to better forecasts of extinction risk and range dynamics [17], especially if the sensitivity of evolutionary adaptation to environmental and demographic conditions can be quantified and incorporated in models of population persistence [18].

At the community level, mesocosms provide an important opportunity to explore and disentangle mechanisms of community assembly and, thus, better establish how climate shifts are likely to affect biodiversity, community structure, and the ecosystem processes that they maintain. Mesocosms can be used to quantify the effect of global warming on species composition and turnover, the strength of biotic interactions, and the distribution of functional traits (e.g., body size), among other ecological processes. This information can be used to parameterize models of local (α) and regional (ϒ) diversity (Fig 1). For example, metacommunity models can potentially be used to explore the likely influence of climate change on connected local community assemblages (i.e., communities linked by dispersal and multiple interacting species) and to improve key theoretical paradigms on how spatial dynamics and local interactions shape community structure [19]. Furthermore, estimates of ecological mechanisms driving temperature-related shifts in species assemblages can be used to test key theories underpinning spatial community ecology, such as temperature-driven body-size reduction at the community level [20], the effect of trophic interaction strengths on food-web structure, and the role of community composition on stability and persistence [7]. Together this will improve forecasts of biodiversity loss and provide crucial information on how to maintain ecosystem processes and services in the face of species loss (Fig 1). Forecasts and theoretical evidence of ecological responses to climate change will be strongest if mesocosms account for a wide range of future climate change scenarios (including variation in extreme events) [13] and potential synergies of drivers of global change (e.g., habitat fragmentation and exploitation) [11].

Theory predicts that climate change will predominantly threaten tropical ectotherms, which are currently living very close to their optimal temperature, while temperate ectotherms, which are living in climates that are currently cooler than their physiological optima, are expected to resist or even benefit from warming [21]. However, Bestion et al. [14] show, using a large-scale outdoor mesocosm experiment, that this generality is by no means universal. Experimental warming of ambient temperature (+ 2°C) increased the juvenile growth rate and reduced the reproduction age of common lizards (*Zootoca vivipara*). However, these temperature-driven enhancements to juvenile and reproductive fitness came at a harmful cost to adult survival. By integrating experimental estimates of survival, growth, and reproduction into population models, Bestion et al. [14] found that even moderate (and very likely) temperature increases for Europe (+ 2°C) will result in regional extinctions of *Z*. *vivipara* at the southern range of their distribution. These results are a far cry from showing that *Z*. *vivipara* will resist or even benefit from climate change, as has been suggested for temperate ectotherms more generally. Even more alarming is the fact that *Z*. *vivipara* is not a physiological specialist with respect to temperature (having a wide-range across Europe and Asia) and therefore not an obvious “at risk” species from climate change [22]. Nevertheless, 21st century climate change is likely to have a strong deleterious effect on its range dynamics, causing regional extinctions that will lead to wide-scale range contraction.

Recent studies have linked human-induced climate change to reduced body size at the population or community level, leading to the suggestion that body-size reduction is a universal response to global warming alongside changes in the phenology and distributions of species [23]. Using a 5-year outdoor mesocosm warming experiment that allowed for natural dispersal, Yvon-Durocher et al. [20] show the exact opposite pattern for phytoplankton communities, tiny organisms that form the basis of food chains in aquatic ecosystems. The researchers warmed artificial ponds containing plankton by 4°C, replicating likely temperature shifts for many of the world’s lakes and rivers in the near future [24]. Warming resulted in more species-rich phytoplankton communities, dominated by larger species. The ecological mechanisms responsible for this somewhat unexpected finding appears to be an increase in top-down regulation of community structure, in which warming systematically shifted the taxonomic composition of phytoplankton towards large-bodied species that are resistant to grazing by zooplankton. Increased biodiversity, due to greater species coexistence, is likely to have resulted from a reduction in competitive exclusion between large (and inedible) phytoplankton, which are inferior competitors for nutrients. Furthermore, warmed mesocosms had higher gross primary productivity due to increases in the biodiversity and biomass of the phytoplankton communities. Together, these findings show that in ecosystems where local extinctions can be counterbalanced by immigration, warming can lead to increases in biodiversity and function and to an increase in mean body size at the community level.

Both studies promise to strongly influence future climate-change ecology research. For example, we now have a stronger understanding of the importance of (1) establishing the impact of climate change on the entire life cycle of a species and using this detailed information to identify populations at risk of extirpation from future global warming and (2) taking a “whole community” multispecies-type approach to predicting the impacts of climate change on biodiversity. More generally, these studies are prime illustrations of how mesocosms can deepen our understanding of the ecological consequences of climate change, often providing surprising yet vital results along the way.

Today's scientists are faced with the task of forecasting how climate change will affect species distributions and species assemblages. A pressing challenge is to develop integrated modelling frameworks that account for all aspects of vulnerability: exposure, sensitivity, and adaptive capacity [4]. Directly accounting for climate-driven changes in survival, persistence, and fitness (sensitivity) can provide improved forecasts of extinction risk [16], yet model predictions rarely account for the demographic and physiological sensitivities of species to prevailing climates. Biological processes underlying adaptation of a species to its environment remain poorly understood. Rare attempts to include evolutionary responses directly in climate-biodiversity models have shown that predictions of vulnerability can be affected by adaptive capacity [15]. Mesocosm experiments are key to meeting this shortfall, providing valuable information on aspects of climate change ecology (e.g., the impact of extreme events on species survival, climate as a driver of phenotypic changes) that cannot be readily assembled from other approaches [13]. Establishing multigenerational mesocosm experiments systematically, using taxa representing a diversity of ecological and evolutionary milieu, and integrating observed demographic and physiological responses into simulation models is likely to strengthen confidence in climate-impact science and improve vulnerability assessments (Fig 1) [17]. This will be particularly so for short-lived taxa that are passively dispersed or with short active dispersal requirements. Developing mesocosm experiments for long-lived, wide-ranging species will be much less feasible.

At the community level, species will not respond equally to climate change. Some may adapt better, and some may track changing climates faster than others. This will affect the structure and dynamics of species interaction networks both by breaking already established interactions and by the appearance of novel interactions [25]. By developing and testing theoretical expectations of climate-driven changes in ecological network structures of communities, mesocosms can be used to improve knowledge of how functional traits can predispose species to range expansion or contraction under shifting climates and their associated effects on community structure and stability, and food web organization and dynamics [13,25]. Mesocosms can also be used to better identify and understand ecological mechanisms that enable spatial habitat structure to buffer communities from the effects of climate change [11]. These types of information are essential if we are to move beyond extrapolating biodiversity loss from species-level models to parameterizing and refining more ecologically realistic multispecies predictive models (Fig 1) [26].

Deriving the full benefits of coupling mesocosm experiments with theory and real-world observations to better predict and mitigate the worst effects of climate change on biodiversity will require an immediate movement away from short-sighted funding strategies. This is because ecological responses to climate change can take multiple generations to be expressed [20]. Furthermore, there needs to a be a more unified approach to the use of mesocosms in climate change research, whereby investigators and funding bodies alike see the benefit of simultaneously replicating experiments across different systems, to establish the generality of results and theory [7]. Doing this will avoid extrapolating from isolated, uncoordinated, and contingent case studies [13]. Lastly, predictions of biodiversity loss from climate change will be improved by adopting a wider range of future climate change scenarios in mesocosm experiments. Future scenarios should include changes in the frequency, duration, and magnitude of extreme events, as well as gradual shifts in average conditions.
